# Heregulin β-1 Induces Loss of Cell-Cell Contact and Enhances Expression of MUC1 at the Cell Surface in HCC2998 and MKN45-1 Cells

**DOI:** 10.1371/journal.pone.0029599

**Published:** 2011-12-22

**Authors:** Rintaro Okoshi, Chung-Li Shu, Sayoko Ihara, Yasuhisa Fukui

**Affiliations:** 1 Institute of Cellular and System Medicine, National Health Research Institutes, Zhunan, Taiwan, Republic of China; 2 Division of Applied Biological Chemistry, Graduate School of Agricultural and Life Sciences, University of Tokyo, Bunkyo-ku, Tokyo, Japan; 3 Laboratory of Signal Transduction, Hoshi University, Shinagawa-ku, Tokyo, Japan; National Cancer Center, Japan

## Abstract

Signal transduction and cell responses after stimulation with heregulin β-1 (HRG) are examined in HCC2998 and MKN45-1 cells, which have been used for a model system to study the formation of signet ring carcinomas, one of poorly differentiated adenocarcinomas. HRG stimulation causes rounding of the cells, responding to HRG. The adherens junction, which is present in the control cells, is disrupted and cell-cell interaction is lost after stimulation. Inhibition of phosphatidylinositol (PI)-3 kinase or p38 MAP kinase blocked this reaction, which indicates that the PI-3 kinase-p38 MAP kinase pathway is required for this reaction. Inhibition of the p38 MAP kinase pathway resulted in immediate restoration of cell-cell interaction. This result indicates that signaling for adherent molecules is strictly regulated by growth factor signaling. Expression of MUC1 at the cell surface is also observed and found to be expressed only after HRG stimulation. The total amount of MUC1 remains unchanged, suggesting that this amount is not due to induction of gene expression but to translocation of MUC1 from the inner membrane to the plasma membrane. This reaction is independent of the cytohesin pathway but dependent on PI-3 kinase activity. In addition to these reactions, HRG stimulates cell growth of both HCC2998 and MKN45-1 cells, depending on the ERK pathway given that the MEK inhibitor abolishes this effect. Therefore, HRG induces various reactions in HCC2998 and MKN45-1 cells by different pathways. These reactions are all related to characteristics of tumors, which implicates that HRG signaling can contribute to the formation of tumors.

## Introduction

Heregulins/neuregulins are growth factors that form a family and present at the apical side of the epithelial cells: their signaling has been implicated to influence cell polarity [1]–[5]. HRG takes ErbB3/HER3 as a receptor [6]. ErbB3 is a member of the EGF tyrosine kinase receptor family but may have lost its enzymatic activity because of a substitution of the amino acid essential for the process [7]–[9]. HRG activates ErbB3 to make heterodimers with other members of the EGF receptor family. The major signaling pathways that are activated after HRG stimulation are suggested to be the phosphatidylinositol (PI) 3-kinase and the ERK pathways [8], [10]. Activation of ErbB3 by making a heterodimer with ErbB2 has been suggested to be related to formation of breast cancers [6], [10]–[14].

Signet ring cell carcinomas are one of the poorly differentiated adenocarcinomas originally found mainly in northeastern Asia as stomach cancers. However, nowadays this kind of cancer is found in various areas and in various organs [15], [16]. Because these cells grow without interaction with other cells and because cells secrete mucins, such as MUC1, to cover the cells, chemical treatment of these carcinomas and related surgery are extremely difficult.

In many of the signet ring cell carcinoma cell lines, the ErbB2/ErbB3 pathway is often constitutively activated by the autocrine loop of ErbB2-ErbB3-Muc4-ErbB2[17], [18], [19]. No further activation of the ErbB2/ErbB3 pathway even if cells are stimulated by HRG. But in the other cell lines, no activation of ErbB2/ErbB3 is observed. As shown in Table 1, it appears that there are two types of signet ring carcinomas: ErbB2/ERbB3 activated and non-activated [20]–[22]. HCC2998 cells are the highly differentiated colon adenocarcinoma cells used as model cells in studies of the formation of signet ring carcinomas [23]. When constitutively activated PI-3 kinase is expressed in the cells, cell-cell contact is lost and secretion or cell surface expression of mucins is enhanced to become very similar to that of signet ring carcinoma cells [17], [23]. To show the effect of HRG is not limited to HCC2998 cells, a gastric adenocarcinoma line MKN45-1 was also used. This cell line derives from MKN45 poorly differentiated adenocarcimona line [24]. While we were culturing these cells, relatively flat cells appeared. This was unusual because poorly differentiated adenocarcinoma cells rarely yield highly differentiated adenocarcinoma cells [23].We cloned these cells and named them MKN45-1. These cells behaved similarly to HCC2998 cells, have been used as a model for signet ring cell carcinomas [23]. However, the regulation of these cell responses is not well understood. Concerning dissociation of the cells, only the contribution of the p38 MAP kinase, which lies downstream of the PI-3 kinase in these cells, is known [18]. In addition, these results were obtained only in tumor cell lines or after expression of a constitutively active mutant. Therefore, the effect of stimulation with the natural ligand on intact cells should be examined precisely.

**Table 1:** Characteristics of signet ring cell carcinoma cell lines^a^.

Cell lines	Status of ErbB2/ErbB3 complex, response to HRG
HSC39	activated (phosphorylated), no further activation
HSC45	not detected, no reaction
HSC58	activated (phosphorylated), no further activation
HSC60	not detected, no reaction
NUGC4	activated (phosphorylated), no further activation
KATOIII	activated (phosphorylated), no further activation

a Status of ErbB2 and ErbB3; and responses to HRG are shown in various signet ring cell carcinoma cell lines. HSC45, HSC58, and HSC60 grow as mixtures with other types of cells and cannot be purified.

In this paper, we demonstrate that HRG can cause loss of cell-cell contact and enhance the secretion of MUC1, a mucin, in HCC2998 and MKN45-1 cells, as well as cause cell growth through different signaling pathways.

## Materials and Methods

### Cells and culture conditions

HCC2998 and MKN45-1 cells were cultured in RPMI1640 medium supplemented with 5% fetal calf serum [23]. In the experiments cells were stimulated with HRG (100 ng/ml), which was purchased from R&D Systems (Minneapolis, MI). HRG was given to the cells every 24 h because its effects last only about that long.

### Antibodies and reagents used in this study

Anti-ErbB3 (C-17) and anti-p38 antibodies were purchased from Santa Cruz Biotechnology Inc. (Santa Cruz, CA). Anti-ErbB2 antibody, CB11, was from Biocare Medicals (Concord, CA). Anti-MUC1 antibodies were from Acris Antibodies GmbH (Herford, Germany) and Millipore (Billerica, MA). Anti-E-cadherin and β-catenin antibodies were from GE Healthcare (London, UK). Anti-ERK, Anti-phosho-ERK, anti-p38 MAP kinase, anti-phospho-p38 MAP kinase, and anti-phospho-ErbB3 antibodies were from Cell Signaling Technology (Danvers, MA).

A potent PI-3 kinase inhibitor, ZSTK474 [25] was a kind gift from Dr. S. Yaguchi (Zenyaku Kogyo Co. Ltd). A MEK inhibitor, PD98059, and a p38 MAP kinase inhibitor, SB202190, were purchased from WAKO Co. Ltd. (Tokyo). We used SB203580 9 (WAKO Co. Ltd.) as a p38 MAP kinase inhibitor. However, the results were always the same as those with SB202190. We therefore present the results of SB202190 in this paper. SecinH3 was from Millipore (Billerica, MA).

### Immunocytochemistry

Cells were fixed with 3.7% formaldehyde. After treatment with PBS with or without 0.2% Triton X100, cells were incubated with primary antibodies for 1 h and visualized with Alexa488 or Alexa594-conjugated second antibodies (GE Healthcare). F-actin was visualized by TRITC-phalloidin (Sigma-Aldrich, St. Louis, MO). Cells were observed by confocal microscopy (Olympus, FV300).

### Western blotting, immunoprecipitation, and biotinylation of the cells

Western blotting was done using PVDF membrane as described before [26]. Immunoprecipitation and biotinylation of the cells were done as described before [19].

### Fractionation of the cells

Cells were suspended in a buffer containing 10 mM Tris-HCl, pH 7.5, 10 mM NaCl, and a proteinase inhibitor cocktail; they were then homogenized in a Dounce homogenizer. After removing the nuclei by low-speed centrifugation, the cytoplasmic fraction was further fractionated into the membrane and the cytosolic fractions by spinning at 45,000 rpm for 60 min.

### Examination of cell growth

1×10^5^ cells were plated in 3.5 cm dishes. Cells were harvested by trypsinization and counted every day.

### Flow cytometry

After fixation with 3.7% formaldehyde, cells were scraped and passed though a syringe to break cell aggregates. After treatment with anti-MUC1 antibody, cells were washed with PBS once and reacted with an Alexa488-conjugated second antibody (GE Healthcare). After washing with PBS three times, the samples were subjected to flow cytometry.

## Results

### HCC2998 cells express ErbB2 and ErbB3, and respond to HRG

**Figure 1:**
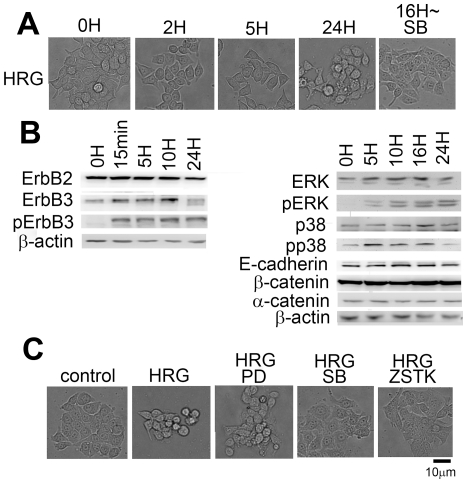
Reaction of HCC2998 cells to HRG stimulation. (**A**) HCC2998 cells were treated with HRG (100 ng/ml) for the indicated time. Cells were observed under the microscope. “∼16 h SB” indicates addition of SB202190 (1 µM) to the culture after incubation with HRG for 16 h. (**B**) HCC2998 cells were treated with HRG (100 ng/ml) for the indicated time. Protein levels and their phosphorylation were analyzed by Western blotting. (**C**) HCC2998 cells were treated with HRG in the presence of PD98059 (PD), SB202190 (SB), or ZSTK474 (ZSTK) for 24 h. Cells were observed under the microscope.

**Figure 2:**
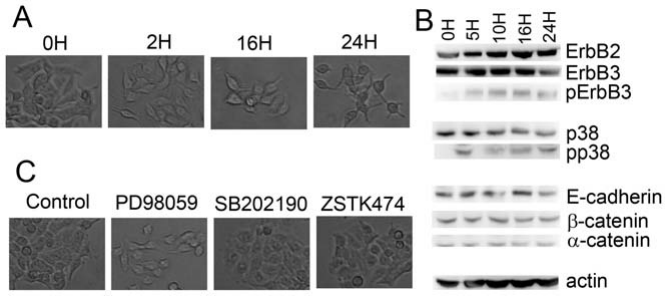
Reaction of MKN45-1 cells to HRG stimulation. (**A**) MKN45-1 cells were treated with HRG (100 ng/ml) for the indicated time. Cells were observed under the microscope. (**B**) HCC2998 cells were treated with HRG (100 ng/ml) for the indicated time. Protein levels and their phosphorylation were analyzed by Western blotting. (**C**) HCC2998 cells were treated with HRG in the presence of PD98059 (PD), SB202190 (SB), or ZSTK474 (ZSTK) for 24 h. Cells were observed under the microscope.

HCC2998 cells were treated with HRG. As shown in Fig. 1A, loss of cell-cell contact was observed starting from a few hours after HRG stimulation. Because cells do not move much, it was hard to tell whether they really dissociated; but rounding of the cells suggested that they may have lost cell-cell contact. Regardless, change of cell shape suggests that these cells responded to HRG. These results were further confirmed using another cell line, MKN45-1 (Fig. 2A). These cells moved a little more than the HCC2998 cells, so dissociation of the cells was obvious. Given these results, the ErbB2/ErbB3 signaling pathway and its outputs were examined. As shown in Fig. 1B, in HCC2998 cells both ErbB2 and ErbB3 were expressed, and phosphorylation of ErbB3 was readily detectable 15 min after stimulation with HRG and continued for at least 24 h. Phosphorylation of ErbB3 was also detectable for at least 24 h in MKN45-1 cells (Fig. 2B). Degradation of ErbB3 was observed in both cell lines after incubation with HRG for 24 h, although phosphorylation of ErbB3 was still detectable, suggesting that, once phosphorylated on tyrosine, some ErbB3 may be degraded. Prolonged activation of ErbB3 may also lessen the level of intact ErbB3. Phosphorylation of ErbB3 has been shown to activate the ERK pathway and the PI-3 kinase pathway, which has been suggested to activate p38 MAP kinase in these cells [18]. Upon activation of ErbB3, tyrosin phosphorylation of ERK and p38 MAP kinase was detected. Unlike the case when the cells are stimulated with other growth factors, these kinases were activated for a long period. This may be due to prolonged activation of ErbB3.

### HRG induces dissociation of the cells

Since expression of dominant-active PI-3 kinase or MKK6 has been shown to induce rounding of the cells, the effect of inhibitors on the pathway that contains these enzymes was tested. As shown in Fig. 1C and 2C, a PI-3 kinase inhibitor, ZSTK474, and a p38 MAP kinase inhibitor, SB202190, blocked the dissociation of the cells; but a MEK inhibitor, PD98059, failed to do so.

**Figure 3:**
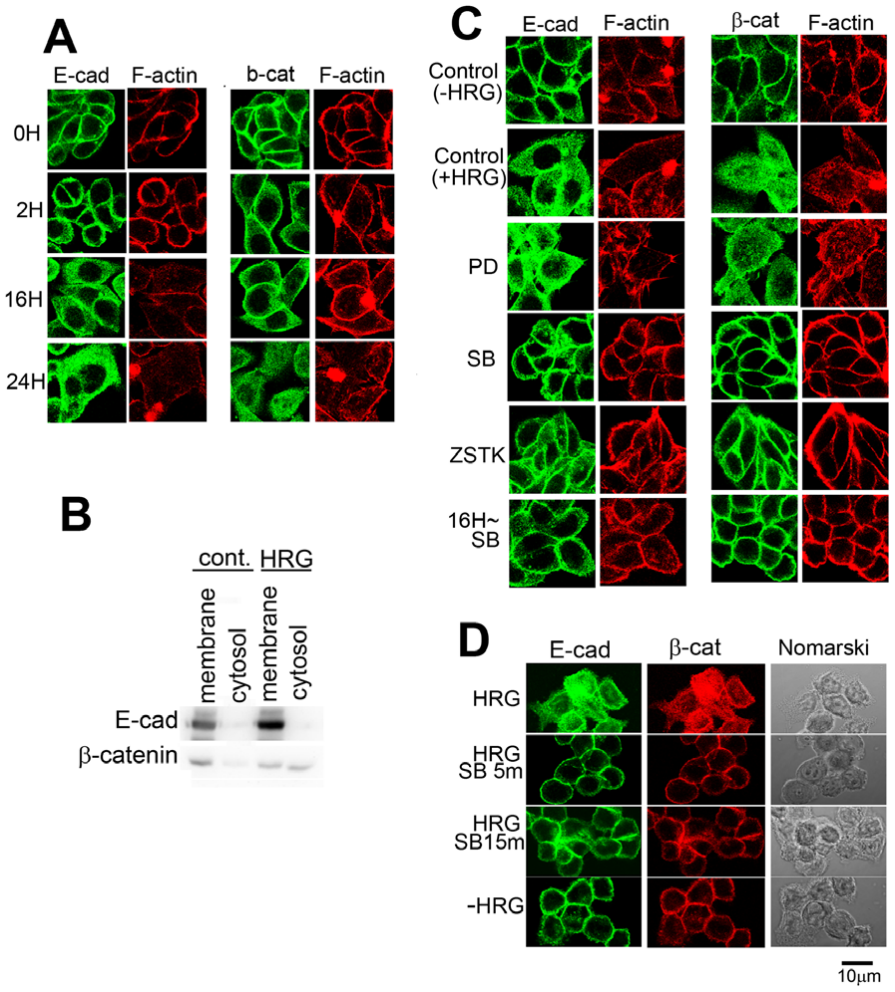
E-cadherin and β-catenin in HCC2998 cells stimulated with HRG. (**A**) HCC2998 cells were stimulated with HRG for the indicated time. E-cadherin or β-catenin was co-stained with F-actin. (**B**) HCC2998 cells were treated with HRG for 24 h. Cytoplasmic fraction of the cells was further fractionated into the membrane and the cytosolic fractions. The membrane fraction was dissolved in the same volume of the sample buffer used for electrophoresis of the cytosolic fraction. The resulting samples were analyzed for the levels of E-cadherin and β-catenin by Western blotting. (**C**) Effect of inhibitors for various signaling molecules (PD98059, 10 µM; SB202190, 1 µM; ZSTK474, 1 µM) on HCC2998 cells treated with HRG for 24 h. E-cadherin or β-catenin was co-stained with F-actin. “16h∼ SB” indicates addition of SB202190 (1 µM) to the culture after incubation with HRG for 16 h and further incubation for 8 h. Sections at about the center of the cells are shown. (**D**) Cells were treated with HRG for 22 h. Then 1 µM SB202190 was added for the indicated time. Cell morphology and localization of E-cadherin was monitored by immunostaining. Sections of the cells at about the middle parts are shown.

**Figure 4:**
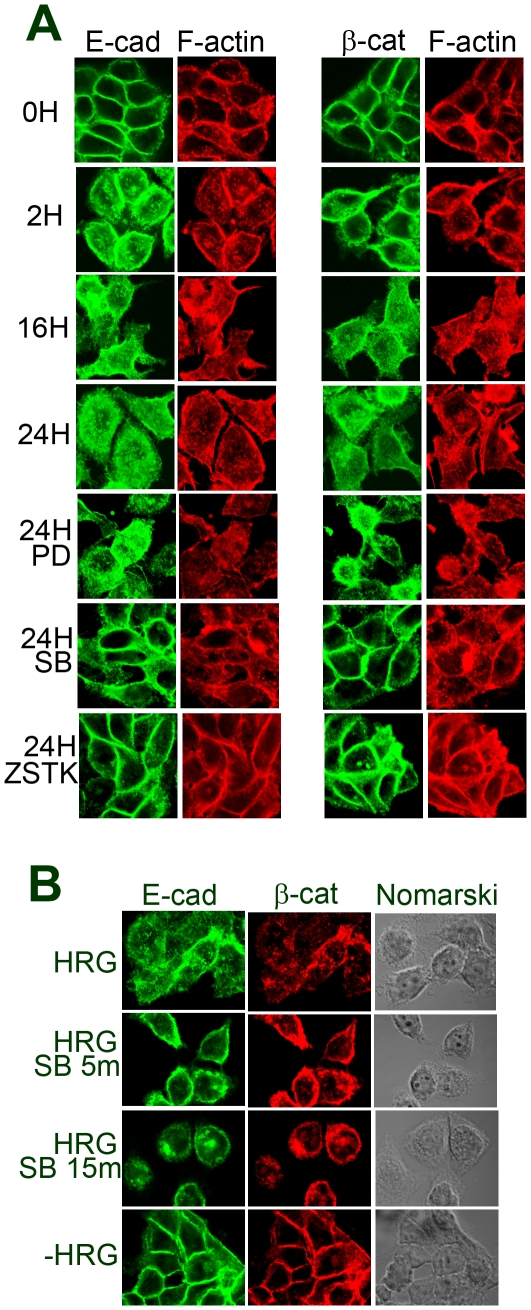
E-cadherin and β-catenin in MKN45-1 cells stimulated with HRG. (A) MKN45-1 cells were stimulated with HRG for the indicated time. E-cadherin or β-catenin was co-stained with F-actin. Effect of inhibitors for various signaling molecules (PD98059, 10 µM; SB202190, 1 µM; ZSTK474, 1 µM) on MKN45-1 cells treated with HRG for 24 h. (B) Cells were treated with HRG for 22 h. Then 1 µM SB202190 was added for the indicated time. Cell morphology and localization of E-cadherin were monitored by immunostaining. Sections of the cells at about the middle parts are shown.

Dissociation of the cells led us to examine the behavior of E-cadherin and β-catenin. In control cells, signals of E-cadherin were readily detectable at the cell-cell contact (Fig. 3A and 4A). F-actin was also accumulated at the cell-cell contact to co-localize with E-cadherin. After incubation of the cells with HRG for 2 h, some of the cells dispersed, and E-cadherin and β-catenin at the peripheral region became slightly fuzzy, suggesting that breakdown of the adherens junctions had already started. These proteins gradually diffused toward inside the cells and finally became even at 24 h after HRG stimulation. F-actin was often left at the peripheral region of HCC2998 cells even after cells were dispersed. In MKN45-1 cells, diffuse distribution of F-actin was seen earlier than in HCC2998 cells. The reason F-actin behaved differently in these cells is not known. During this procedure, expression levels of E-cadherin, α-catenin, and β-catenin remained unchanged (Fig. 1B and 2B).

Movement of the proteins was also monitored by a biochemical method in HCC2998 cells. Cytoplasmic fraction was fractionated, and distribution of E-cadherin and β-catenin was examined. As shown in Fig. 2B, E-cadherin remained in the membrane fraction, suggesting that it remains on the inner membrane even if internalized. However, a considerable amount of β-catenin moved to the cytosol, suggesting that β-catenin was released from the adherens complex, which consisted of E-cadherin and three types of catenins. These results suggest that the complex can be broken down after internalization.

Behavior of E-cadherin, β-catenin and F-actin in the cells cultured in the presence of PI-3 kinase, MEK, and p38 MAP kinase inhibitors was examined (Fig. 3C and 4A). In both cell lines, inhibition of the PI-3 kinase or the p38 MAP kinase blocked dissociation of the cells and kept the structure of E-cadherin, β-catenin, and F-actin intact even after treatment with HRG, suggesting that the PI-3 kinase-p38 MAP kinase pathway regulated the adherens junction. PD98059 was not effective in doing so. It is interesting to note that even if cell-cell contact was lost by treatment with HRG for 16 h, the cell-cell contact was restored within 8 h by addition of SB202190 even in the presence of HRG (Figs. 1A and 3C). These results suggest that adherens junctions are regulated reversibly. Reaction of the cells just after SB202190 treatment was examined precisely.

E-cadherin was diffusely distributed by treatment of the cells with HRG for 22 h, which moved to the plasma membrane leaving almost no signal in the cytosol within 5 min accompanied by rounding of the cells. In the case of MKN45-1 cells, they immediately started to bind to the dish and within 15 min cytosol became turbid again. The reaction of the cells to the signaling of the p38 MAP kinase pathway was quite fast (Fig. 3D and 4B). However, how long it takes to make cell-cell contact is not known.

### HRG treatment of HCC2998 cells induces expression of MUC1 at the cell surface

**Figure 5:**
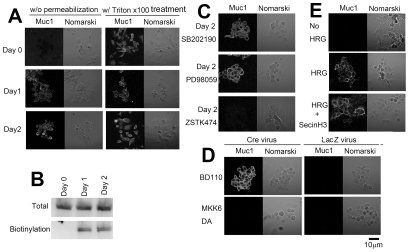
Expression of MUC1 at the cell surface after stimulation with HRG. (**A**) HCC2998 cells were stimulated with HRG for the indicated time. A second dose of HRG was given after incubation for 24 h for the 2-day cultures. Muc1, with or without permeabilization, was stained with anti-MUC1 antibody. On the right sides, Nomarski view of the same cells are shown. (**B**) HCC2998 cells were stimulated with HRG for the indicated time. Total MUC1 levels and MUC1 at the cell surface (biotinylation) were analyzed by Western blotting. Detecting MUC1 expressed at the cell surface required 50 times more protein than was used for simple Western blotting. (**C**) Effect of inhibitors for various signaling molecules on MUC1 expression at the cell surface was examined. HCC2998 cells were cultured for 2 days in the presence of HRG. (**D**) HCC2998 cells bearing inducible genes for dominant active PI-3 kinase (BD110) and MKK6 were used [18], [23]. They were infected with adenovirus bearing Cre recombinase to induce the gene. Adenovirus bearing LacZ was used as a control. (**E**) Experiments similar to **C** were done using SecinH3 (20 µg/ml).

**Figure 6:**
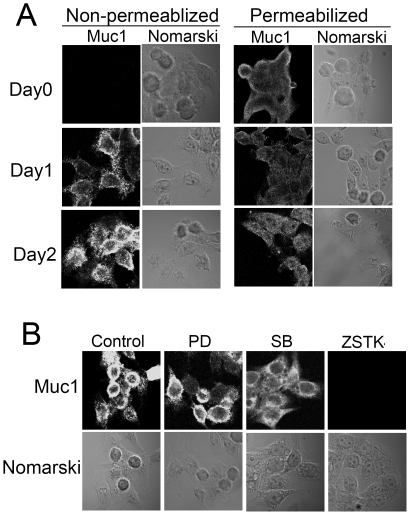
Expression of MUC1 at the cell surface after stimulation with HRG. (**A**) MKN45-1 cells were stimulated with HRG for the indicated time. A second dose of HRG was given after incubation for 24 h for the 2-day cultures. Muc1, with or without permeabilization, was stained with anti-MUC1 antibody. On the right sides, Nomarski views of the same cells are shown. (**B**) Effect of inhibitors for various signaling molecules on MUC1 expression at the cell surface was examined. MKN45-1 cells were cultured for 2 days in the presence of HRG.

**Figure 7:**
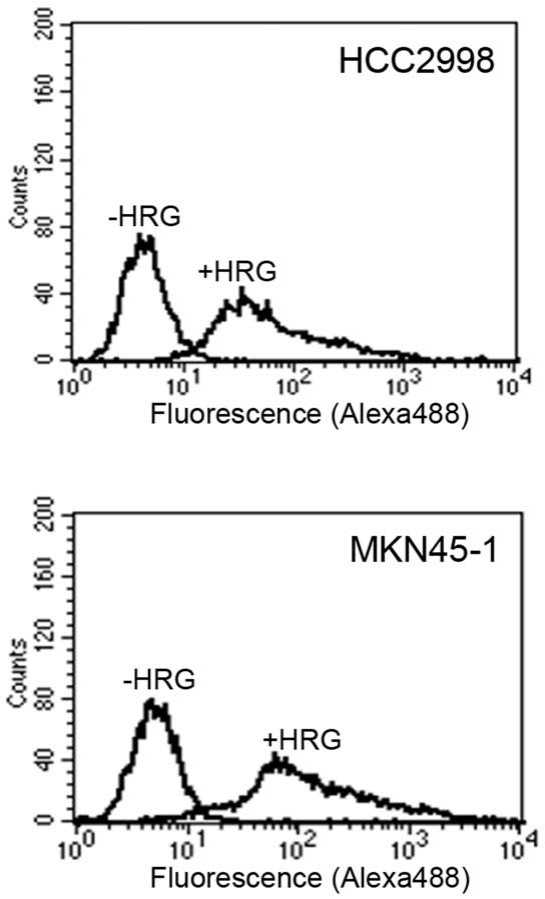
Flow cytometry of HCC2998 and MKN45-1 cells. HCC2998 and MKN45-1 cells treated with or without HRG for 2 days. A second dose of HRG was given 24 h after the first dose. Cells were fixed, dispersed, and stained with an anti-MUC1 antibody, followed by the second antibody conjugated with Alexa488. The resulting cells were subjected to flow cytometry.

Because enhancement of mucin secretion has been observed in signet ring carcinoma cells, behavior of MUC1, one of the mucins, has been examined. When cells were stained with anti-MUC1 antibody without permeabilization, no MUC1 was detected, suggesting that MUC1 was not expressed at the cell surface (Fig. 5A and 6A). MUC1 was present inside the cells because it was detected after permeabilization (Fig. 5A). This was confirmed by Western blotting (Fig 5B). After treatment of the cells with HRG for 1 day, presence of MUC1 at the cell surface became visible (Fig. 5A). Biotinylation of the protein at the cell surface followed by precipitation with avidin-beads in combination with Western blotting supported this idea (Fig. 5B). This was further confirmed by flow cytometry (Fig. 7). In non-stimulated HCC2998 and MKN45-1 cells very low level of signals were detected. In contrast, after two days of stimulation of the cells with HRG, significant levels of the signals were detected, suggesting that MUC1 is expressed at the cell surface. The total MUC1 level remained constant during two days of HRG treatment (Fig. 5B). This reaction was inhibited by ZSTK474 but not by SB202190 (Fig. 5C and 6B). Expression of a constitutively activated PI-3 kinase, BD110 [23], induced expression of MUC1 at the cell surface; but that of activated MKK6 failed to do so (Fig. 5D). These results suggest that translocation of MUC1 is dependent on PI-3 kinase activity but also that the signaling pathway is distinct from that for regulation of adherens junction.

To see the involvement of cytohesins in the translocation of Muc1, the effect of SecinH3, a cytohesin inhibitor, was examined. As shown in Fig. 5E, SecinH3 did not inhibit translocation of Muc1, suggesting that cytohesins were not involved in translocation of Muc1.

### The ERK pathway may be important for cell growth

**Figure 8:**
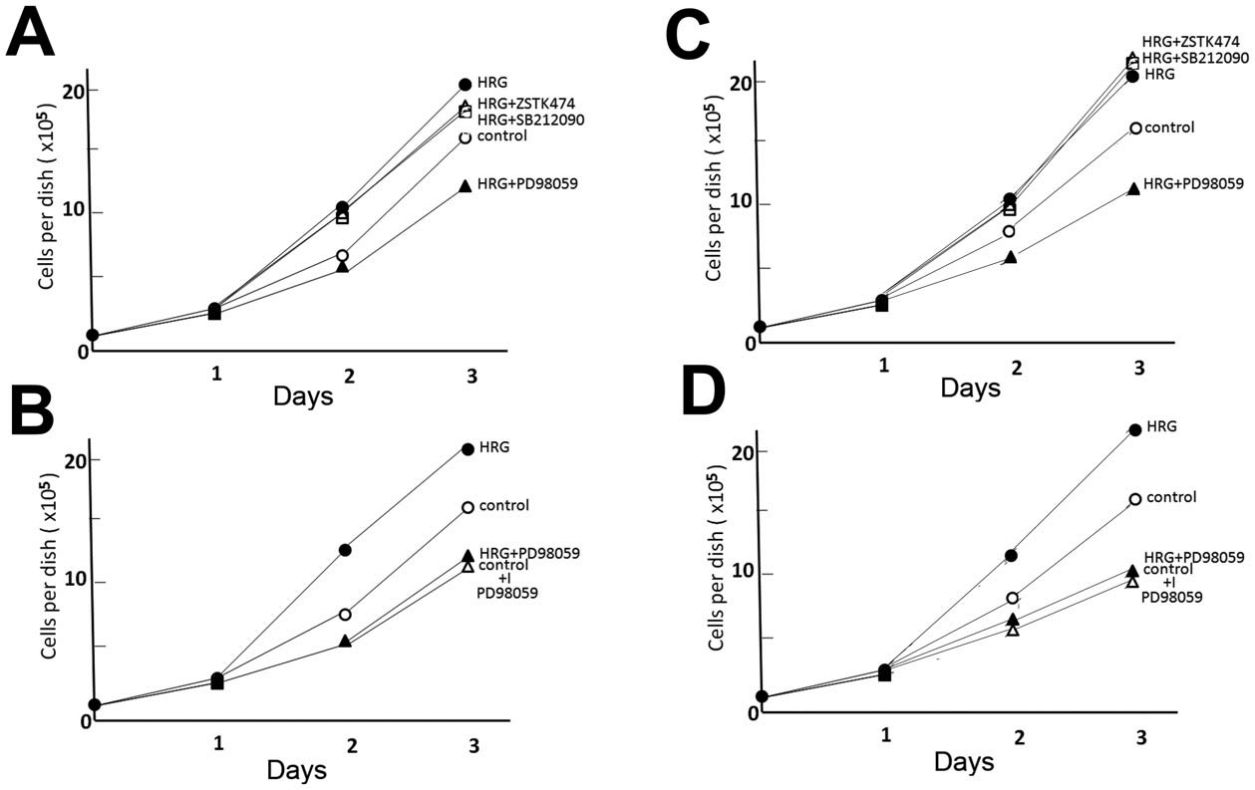
Effect of inhibitors for signaling molecules on growth of HCC2998 and MKN45-1 cells. (A and B) HCC2998 cells were plated in 3.5 cm dishes at a cell density of 1×10^4^ and stimulated with HRG every day in the presence or absence of drugs. Cells in the dishes were counted. (C and D) Similar experiments were done with MKN45-1 cells. Experiments were done three times and plotted on the graph. The standard deviations for each point were less than 4%. Control: without HRG treatment.

The effect of HRG on cell growth was examined. HRG stimulation of HCC2998 and MKN45-1 cells enhanced cell growth (Fig. 8). This was slightly inhibited by inhibition of the PI-3 kinase or p38 MAP kinase in HCC2998 cells but not in MKN45-1 cells. In contrast, inhibition of MEK severely inhibited cell growth. Even in the presence of HRG, the growth rate was lower than the control cells. Because inhibition of MEK decreased growth of control cells as well, it is likely that MEK contributes to cell growth in HCC2998 and MKN45-1 cells and that complete inhibition of MEK results in a very low growth rate (Fig. 8). Super activation of MEK by HRG stimulation may enhance the cell growth greatly.

## Discussion

**Figure 9:**
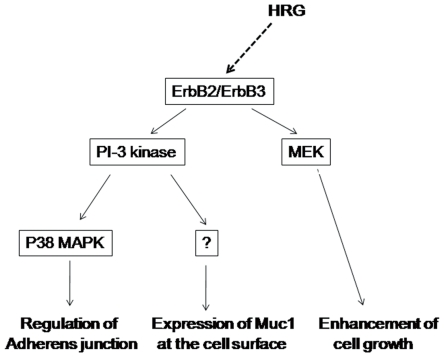
Signal transduction of HRG in HCC2998 cells. HRG induces various cell responses through different signaling pathways.

As demonstrated in this paper, HRG induces various cell responses. It is interesting that each signaling pathway activated by HRG stimulation correlates with different cell responses (Fig. 9). For instance, p38 MAP kinase is likely to be involved only in cell-cell dissociation and not in cell growth or translocation of MUC1. On the other hand MEK was not involved in cell-cell dissociation or translocation of MUC1. The PI-3 kinase pathway may branch into at least two pathways: one for regulation of cell-cell contact through p38 MAP kinase, the other for translocation of mucins. Therefore, each pathway is likely to have an independent role. Downstream signaling of MEK remains to be studied.

How tight and adherens junctions are formed has been widely studied [27]–[33]. However, how these junctions are regulated has not yet received much attention. These junctions must be regulated in some way because they are disrupted in cases such as cell division. Therefore, some systems must regulate these junctions. The p38 MAP kinase cascade might be one of them. Dissociation of the cells by activation of the p38 MAP kinase cascade takes several hours. However, movement of E-cadherin and β-catenin for triggering re-association after shutting off the p38 MAP kinase pathway was very quick. Therefore, the p38 MAP kinase pathway could directly modulate the adherent molecules. The fact that the p38 MAP kinase was activated for at least 24 h after HRG treatment to induce scattering supports this idea.

Cytohesins are PI-3 kinase dependent factors for transportation of proteins. Translocation of MUC1 is suggested to be independent of this factor because SecinH3, a cytohesin inhibitor, did not block the translocation. The fact that overexpression of ARNO or an ARNO mutant incapable of binding to phosphatidylinositol trisphosphate did not alter the translocation supports this idea (data not shown). Therefore, there must be a cytohesin independent pathway that carries out translocation of MUC1. In signet ring cell carcinoma cells, cells are always covered by mucins without any stimulation. It is likely that switch of this unknown pathway for translocation of mucins to the plasma membrane is constitutively on because of constitutive activation of the ErbB2/ErbB3 pathway.

The cell responses mentioned above are all related to malignant transformation of the cells. Therefore, it is conceivable that the ErbB2/ErbB3 pathway can contribute to formation of malignant tumors. Indeed, ErbB2 has been identified as an oncogene [34], and anti-ErbB2 antibody has been used as an anti-tumor drug [35], [36], although ErbB3 is not identified as an oncogene because it may lack enzyme activity. How HRG contributes to the activation of the ErbB2/ErbB3 pathway in vivo is not well understood. Further study may be required to grasp the whole picture of ErbB2/ErbB3-dependent tumors.
